# Direct Transfer of Viral and Cellular Proteins from Varicella-Zoster Virus-Infected Non-Neuronal Cells to Human Axons

**DOI:** 10.1371/journal.pone.0126081

**Published:** 2015-05-14

**Authors:** Sergei Grigoryan, Michael B Yee, Yair Glick, Doron Gerber, Eldad Kepten, Yuval Garini, In Hong Yang, Paul R. Kinchington, Ronald S. Goldstein

**Affiliations:** 1 Mina and Everard Goodman Faculty of Life Sciences, Bar-Ilan University, Ramat-Gan, Israel; 2 Departments of Ophthalmology, Microbiology and Molecular Genetics, University of Pittsburgh, Pittsburgh, Pennsylvania, United States of America; 3 Department of Physics, Bar-Ilan University, Ramat-Gan, Israel; 4 Department of Biomedical Engineering, Johns Hopkins University, School of Medicine, Baltimore, Maryland, United States of America; 5 SiNAPSE National University of Singapore, Singapore, Singapore; University of California Riverside, UNITED STATES

## Abstract

Varicella Zoster Virus (VZV), the alphaherpesvirus that causes varicella upon primary infection and Herpes zoster (shingles) following reactivation in latently infected neurons, is known to be fusogenic. It forms polynuclear syncytia in culture, in varicella skin lesions and in infected fetal human ganglia xenografted to mice. After axonal infection using VZV expressing green fluorescent protein (GFP) in compartmentalized microfluidic cultures there is diffuse filling of axons with GFP as well as punctate fluorescence corresponding to capsids. Use of viruses with fluorescent fusions to VZV proteins reveals that both proteins encoded by VZV genes and those of the infecting cell are transferred in bulk from infecting non-neuronal cells to axons. Similar transfer of protein to axons was observed following cell associated HSV1 infection. Fluorescence recovery after photobleaching (FRAP) experiments provide evidence that this transfer is by diffusion of proteins from the infecting cells into axons. Time-lapse movies and immunocytochemical experiments in co-cultures demonstrate that non-neuronal cells fuse with neuronal somata and proteins from both cell types are present in the syncytia formed. The fusogenic nature of VZV therefore may enable not only conventional entry of virions and capsids into axonal endings in the skin by classical entry mechanisms, but also by cytoplasmic fusion that permits viral protein transfer to neurons in bulk.

## Introduction

Varicella Zoster Virus (VZV) is a ubiquitous pathogenic alphaherpesvirus, causing varicella upon primary infection and Herpes zoster (shingles) following reactivation from a latent state that was established in sensory and autonomic neurons upon primary infection. VZV is a highly fusogenic virus, and productively infected cells frequently form multinucleate syncytia consisting of fused cells. These syncytia are present not only in culture, but also in human skin and ganglionic tissues obtained as pathological specimens from patients with disease or following experimental VZV infection of tissues after their grafting into SCID-hu mice [1]. Fusion of VZV-infected cells is considered to be a consequence of the surface presentation of virally expressed glycoproteins that accumulate in membranes destined for incorporation into the virion envelope. VZV glycoproteins in the viral envelope mediate virus binding to susceptible cells, and fusion permits entry of the virion at the plasma membrane or into the intracellular cytoplasmic space following endocytosis [2]. Fusion may occur between cells expressing viral glycoproteins on their cell surface and other infected cells, as well as with adjacent uninfected cells. This permits spread of virus to susceptible cells without requiring extracellular release of virions. VZV glycoproteins, gB and gH-gL contribute to the fusogenic phenotype. For example, gH and gL coexpression is fusogenic and leads to formation of polykarya (reviewed in [3–4]) and gI has been shown to be required for polykaryon formation in skin and T-cells transplanted to SCID mice (reviewed in [5]).

The establishment of VZV latency following infection of neurons during varicella is thought to occur by two mechanisms [6]. One route is hematogenous delivery of virus by VZV infected T-cells infiltrating peripheral ganglia, which transfer virus to neurons, either directly or to non-neuronal cells of the ganglia that then transfer virus to neurons following a presumably limited ganglionic replication [7]. The second route is the infection of axonal terminations in the skin, where they come into contact with VZV in vesicles or infected fibroblasts/keratinocytes [8]. While neurite infection by VZV through receptor mediated fusion and/or endocytosis is likely to occur, it is also possible that infected cells of skin may fuse to neuronal processes. Such cell to axon fusion would permit the delivery of proteins and virions generated in the infecting cell directly into the neuron.

Recent cellular and molecular studies geared towards unravelling the mechanisms of VZV neuronal infection have evolved from the use of differentiated neurons derived from human stem cells ([9–11]). We have previously shown that human embryonic stem cells (hESC)-derived neurons can be infected with VZV and support in vitro productive replication [9]. These hESC-derived neurons have also been used to demonstrate and visualize axonal infection and retrograde axonal transport of VZVGFP labeled capsids in compartmentalized microfluidic chambers. Axonal infections eventually resulted in soma compartment viral replication and spread of infection [12]. In the course of these studies, we observed that in addition to transport of punctate GFP-labeled structures, some axons were rapidly filled with GFP. We now have studied this phenomenon and find that both viral-encoded proteins and non-viral encoded proteins produced in VZV-infected input cells can rapidly enter axons of hESC-derived neurons, apparently as the result of cytoplasmic transfer following fusion. Using fluorescence recovery after photobleaching (FRAP) we obtained evidence supporting this mechanism. FRAP also allowed us to calculate the approximate diffusion rate of GFP and a VZV protein in the axons. In addition, we obtained immunocytochemical evidence supporting fusion of neurons into syncytia of VZV-infected cells.

## Materials and Methods

### Cells and viruses

Human embryonic stem cells (hESC) H9 (WA09; US National Stem Cell Bank) were maintained on mitotically-inactivated human foreskin fibroblasts monolayer in NutriStem medium (Biological Industries, Israel). Medium was changed every other day and cultures were passaged once a week.

Human hESC-derived neurons were generated from neural precursors using PA6 stromal cell co-culture as described previously [12]. Clusters of neural precursors were manually cut from the co-cultures and plated in glass bottom petri dishes (35mm, SPL LifeSciences, Korea), 24-well plates or compartmentalized microfluidic chambers (see below) using protocols described previously [12]. Terminal neuronal differentiation was at least 10 days after plating of the precursors, and medium was changed every other day.

A line of MeWo melanoma cells was derived that stably and constitutively expressed GFP. Cells were transfected with linearized pEGFP-C1 (Clontech Inc, USA) and then selected in media containing 1000ug/ml of G418. Cells used in this study were derived from a clone obtained following cell sorting of the primary G418 resistant population, and GFP-positive cells were maintained in medium containing 400μg/ml of the antibiotic. No institutional ethics review board permission was required in either of the participating research institutes for the use of the standard MeWo, ARPE19 or Vero cell lines that are readily available from culture collections such as the ATCC. The experiments on hESC line H9 were performed at Bar-Ilan University, where there is no requirement for ethics review board permission for use of human embryonic stem cells.

VZV driving GFP expression under the constitutive promoter SV40 (VZV-GFP) was described previously [13]. Generation of VZV expressing mRFP/EGFP as fusions to ORF66/ORF10/ORF23/ORF62 have been described previously [14]. VZV was grown in MeWo or ARPE19 cells in DMEM + 10% fetal calf serum (Biological Industries, Israel), with added penicillin and streptomycin. Infection of neuronal cultures was performed in a cell-associated manner using freshly defrosted aliquots of VZV-infected MeWo or ARPE cells plated into the axonal compartment of the microfluidic chamber system (about 1.2 x 10^5^ cells) or glass-bottom culture dish (about 2 x 10^5^) in neurogenic medium. Each experiment was performed 2–5 times with at least triplicates for each experimental condition. Protein synthesis inhibition was performed by seeding MeWo cells in axonal compartments in the presence of 100mg/ml cycloheximide (Sigma) for the duration of the co-culture period.

Cell-free VZV was purified and concentrated using the protocol described previously [14–15]. In experiments where MeWo cells were infected in a cell-free manner, VZV-66RFP was applied at a concentration of 6 x 10^3^ PFU/ml in wells of a 6-well plate using “spinoculation” at 1000g for 10 min at RT. Infections of MeWo cells with cell-free HSV-1 were performed using the same protocol as for VZV. HSV1 expressing mCherry fused to VP26 was a kind gift of Prashant Desai (Johns Hopkins University, Baltimore, MD). HSV1 was propagated in Vero cells.

### Compartmentalized microfluidic chambers

**Fig 1:**
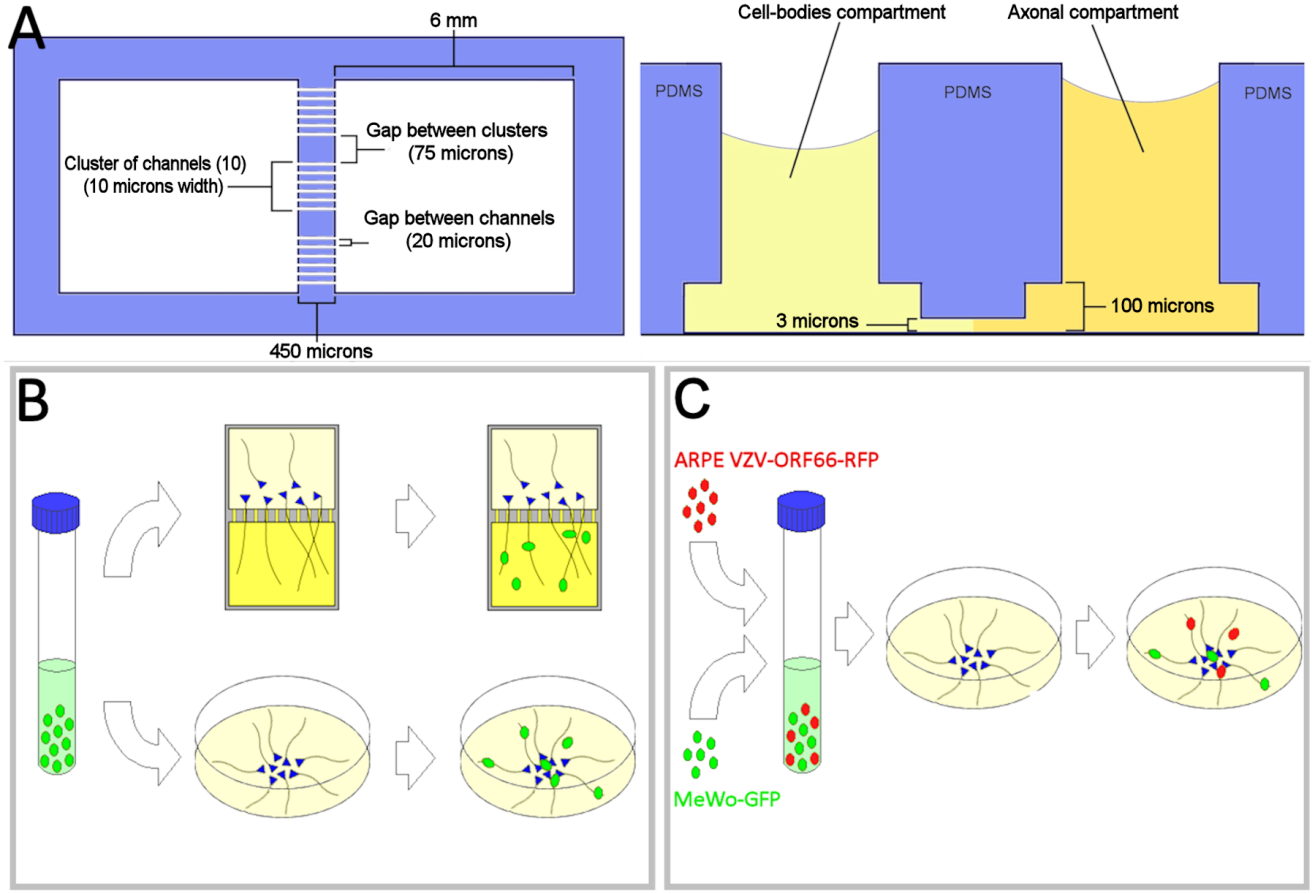
Schematic representations of the microfluidic chambers and experimental methods. (A) Top view and cross-section of a compartmentalized microfluidic chamber. Two identical square chambers (6mm) are connected by 100 microchannels (10μm width, 450μm length and 3μm height) arranged in clusters of 10 with gaps of 20μm between the channels and 75μm between the clusters. A cutout in the silicone layer with a height of 100μm was added to allow seeding neural precursor spheres close to the channels. Differentials of volume and NGF were established between the chambers to attract axons from the soma to the axonal compartment. When the experiment was initiated by seeding VZV-infected cells in the axon compartment, the volume differential was reversed to block diffusion of virions from the axonal to cell-body compartment. (B&C) Schematic representation of experimental methods of cell-associated neuronal infection by VZV. Blue triangles represent neuronal cell-bodies and green or red ovals represent GFP or RFP-expressing non-neuronal cells. In the upper row of B, axons were infected by plating input cells into the axonal compartment of microfluidic chambers. In the lower portion of B, VZV-infected input cells were seeded onto cultures of differentiated hESC-derived neurons. (C) Diagram depicting experimental method used to demonstrate fusion of neurons with non-neuronal cells in syncytia. VZV66RFP infected ARPE cells and GFP-expressing MeWo cells were mixed together and plated on differentiated neurons in glass-bottom culture dishes.

Microfluidic devices of two types were used, with identical results. The first type was that used in previous studies ([9],[12]). The second type was an improved design based on our previous experience (Fig 1A).

The new devices contain 100 channels of 3μm height, 10μm width and 450μm length, separating two square chambers with sides of 6mm. Each layer of the device was reproduced as a chrome mask at 40,000 dpi (Fineline-Imaging USA). The first layer was made using 4” silicon wafers by spin coating SU-8 2005 (MicroChem, USA) at 500 rpm for 10s initially followed by 4000 rpm for 60s yielding a substrate height of around 3–5μm. The molds were baked at 65°C for 2 min and 95°C for 5min. Next, the wafers were exposed to UV light for 3s on the mask aligner, followed by post-exposure baking at 65°C for 1 minute and 95°C for an additional 3 minutes. The wafers were then developed in PGMEA developer (KMG, UK) for 3 minutes followed by an isopropanol wash. The second layer was generated by spin coating SU-8 2050 (MicroChem USA), on developed molds at 500 rpm for 10s followed by 1500 rpm for 60s yielding a substrate height of around 50–75μm. The molds were baked at 65°C for 3 minutes and 95°C for 10 minutes. Next, the second mask was aligned to the wafers and exposed for 10s on the mask aligner, followed by a post-exposure bake series of 65°C for 2 minutes and 95°C for 5 minutes. The wafers were then developed in PGMEA developer (KMG, UK) for 4.5min followed by an isopropanol wash. The microfluidic devices were fabricated in silicone molds from a 10:1 mixture of silicone based elastomer polydimethylsiloxane (PDMS, SYLGARD 184, Dow Corning, USA). The molds were first exposed to chlorotrimethylsilane (TMCS, Sigma-Aldrich, USA) vapor for 10 min to promote elastomer release after the baking steps. The silicone was degassed and baked for 30 minutes at 80°C. The silicone devices were peeled from the mold and access holes punched. Devices were attached to glass-bottom culture dishes or glass coverslips after treatment in a plasma generator for 90s. The complete chambers were sterilized using 70% ethanol and air-dried in a sterile environment.

### Immunocytochemical staining

Immunostainings were carried out as detailed previously [16]. Primary antibodies used were: mouse anti-medium neurofilament subunit (NF-M, DSHB, USA 2H3, 1:15), rabbit anti-heavy neurofilament subunit (NF-H, Sigma-Aldrich, N4142, 1:1000) and mouse anti-Islet-1 homeobox (Isl-1, DSHB 40.2D6, 1:5). Secondary anti-mouse antibodies were conjugates of Alexa-488 (1:500), Alexa-647 (1:250) and 594 (1:2000) (Jackson, USA).

### Live imaging and confocal microscopy

Live cultures were viewed and photographed with a Jenoptiks ProgRes MFcool digital camera (Jena, Germany) on an Olympus IX81 inverted microscope. Long-term live imaging experiments were performed with a Zeiss Observer Z1 imaging system using a Plan Apochromat ×20/0.8 DIC objective and Hamamatsu digital camera or an Olympus IX81 with a LCPlanFLN x20/0.45 phase contrast objective and an Olympus XM10 camera. Confocal imaging was performed using an Olympus FV-1000 confocal microscope, with an UPlanSApo x63/1.35 oil immersion objective. Nikon Intensilight, Lumencor Sola or Thorlabs Plasma solid-state light sources were used for fluorescence illumination. During acquisition of the movies, cultures were maintained in 5% CO2, 37°C and 95% humidity. Zeiss ZEN blue edition and Olympus CellSens software were used for recording movies and Jenoptiks software for recording images.

Quantification of the percentage of fluorescently-filled axons in infected cultures was carried out by counting of axons in micrographs taken from ten arbitrarily-chosen microscopic fields from three independent experiments. The total number of axons was determined in phase-contrast images, and the number of GFP+ axons from fluorescence micrographs of the same microscopic field.

### FRAP methods and data analysis

Experiments using fluorescence recovery after photobleaching (FRAP) were performed using an Olympus FV-1000 confocal system with an UPlanSApo x63/1.35 oil immersion objective. A multi-wavelength argon laser 800AL (225mW, National Laser) was used at 50mW power during the experiments for 488nm excitation and a HeNe laser was used at full power for 561nm excitation. The imaged area was cropped to 300x200 pixels (27.5x41.3μm) and then digitally zoomed x3.

100 images were collected; the first 5 images were of pre-bleached cells and the other 95 of the same field after bleaching with a sampling rate of about 0.47 sec/frame in one-way mode. Axons were photobleached for 1 sec and MeWo cells for 30 sec. Images were analyzed using ImageJ software, and normalization was performed according to the function:

In = ((Im − Ibg)/((It-Ibg)/I0))/Ipb

where In is normalized intensity, Im—measured intensity at time x, Ibg—mean background intensity at time x, It—intensity of another object in the monitored area in time x, Ipb—intensity of the pre-bleached cell and I0 is an intensity of another object in the monitored area (reference area) at time 0. The function normalizes the data, reducing the effect of the laser fluctuations and scanning-caused bleaching during the recovery phase [17].

The concentration of bleached fluorophores is given at first approximation in [18]:

fd(t) = A(1-e-a^2/(4Dt)) + B 

Where A and B are the mobile and immobile fractions respectively, D is the diffusion rate and a^2 is the bleached area. Thus the fluorescence of the bleached area is given by (using A+B = 1):

fd(t) = Ae-a^2/(4Dt)

However, in order to determine the diffusion coefficient of GFP in the bleached areas we used an equation described by [18] with a generalization for one and two dimensional diffusion for axonal GFP:

$${\text{D = (r}}_{\text{n}}{}^{\text{2}}{\text{+ r}}_{\text{e}}{}^{\text{2)}}{\text{/d4t}}_{\text{1/2 }}$$

Where d is the dimension of the diffusion and r_e_^2^ is the effective bleached area. The effective bleached area was determined by fitting the difference profile of pre- and post-bleached images to a Gaussian curve.

## Results

### Axons are rapidly filled with fluorescent proteins after contact with VZV-infected non-neuronal cells in compartmented chambers

**Fig 2:**
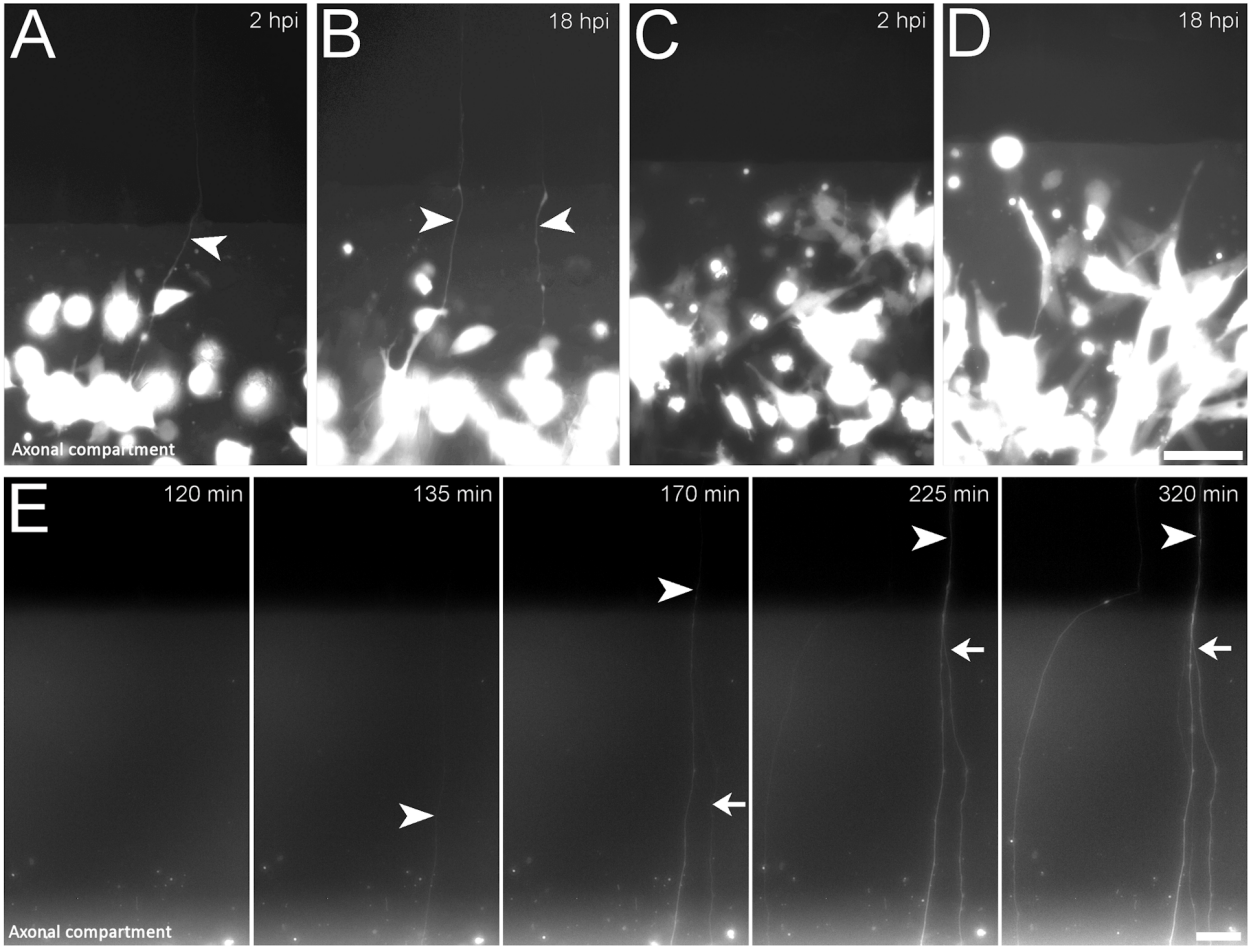
Rapid transfer of protein from VZV-infected MeWo cells to axons. VZV-GFP-infected MeWo cells were plated in the axonal compartments of microfluidic chambers. The cultures were monitored at the junction of the axonal compartment (lower part of each image) and the microfluidic channel (upper half of each image). By 2 hpi, some axons were diffusely filled with GFP (arrowhead in A). (B) The same compartment at 18 hpi, showing additional GFP-filled axons. C & D depict a chamber where MeWo-GFP cells not infected with VZV were plated into axonal compartments at 2 and 18hpi respectively. Even at 4 dpi, no GFP+ axons were observed (not shown). (E) Time series of images of axons in microfluidic channels 2–6h after plating VZV-GFP infected MeWo cells with hESC-derived axons. At 135 minutes pi a GFP-filled axon was first observed (arrowhead). The direction of GFP filling was from bottom to top, i.e., toward the cell-bodies compartment (top of images) since at 170m pi, the same axon displayed GFP fluorescence throughout the microscopic field (3rd panel of E). At this point in time, another GFP-labeled axon (arrow) had appeared in the field. A few hours later, an additional GFP-filled axon appeared in this microscopic field. Scale bars: 50μm. Cell-bodies are “overexposed” in panels A–D in order to visualize the thin and therefore more weakly fluorescent axons. The time-lapse movie of this experiment is S1 Movie.

In our previous studies of retrograde transport of VZV-GFP23 in human axons using compartmentalized microfluidic chambers, we observed rapid appearance of fluorescence in axons after cell-associated infection in the axonal compartment, well before productive infection was expected to be initiated in the cell body compartment [9]. In order to first confirm the rapid appearance of GFP in axons after contact with VZV-infected non neuronal cells with a different recombinant virus, we seeded MeWo cells infected with VZV-GFP, a recombinant virus that expresses GFP driven by an independent SV40 promoter [13], into the axonal compartment of microfluidic chambers (Fig 1A) and monitored the cultures for GFP fluorescence (Fig 1B). Some axons became diffusely labeled with GFP as soon as 2 hours after seeding input cells (hours post infection, hpi), which is a little more than the minimum time needed for newly-thawed infecting cells (see methods) to attach to the substrate (Fig 2A). By 18 hpi, considerably more GFP-positive axons were found in the axonal compartment, indicating that the process is time-dependent (Fig 2B). Importantly, no GFP+ cells were observed in the cell-body compartments of the microfluidic chambers at this time. This is consistent with our observations that retrograde transport, initiation of infection and expression of viral proteins in the neuronal somata require at least 24 hours in this experimental system ([9],[12]). In addition, the direction of the filling of the axons with fluorescence was always from the distal portion of the axon towards the channels leading to the cell bodies. The lack of GFP in the cell body compartment and the retrograde direction of the filling of the axons demonstrate that the source of GFP in the axons was exclusively in the axonal compartment and was not due to replication of the virus, synthesis of GFP in the cell bodies and anterograde diffusion.

In order to estimate the proportion of axons that were filled diffusely, we counted the number of GFP-fluorescent and non-fluorescent axons (using phase contrast) at 18hpi. About 3.5% of 940 axons were diffusely filled with GFP (quantitation of 10 microscopic frames pooled from 3 independent experiments) at this time point. Since not all axons in the culture were in contact with infected MeWo cells, we cannot estimate what percentage of contacts between infected cells and axons resulted in appearance of fluorescent protein. Importantly, when naïve, uninfected GFP-expressing MeWo cells were seeded on axons, no GFP-positive axons were found in control chambers (Fig 2C and 2D).

In order to further characterize the filling of the axons with GFP, time-lapse movies were made in axonal compartments of microfluidic chambers into which VZV-GFP-infected MeWo cells had been seeded (S1 Movie, Fig 2E). Again, analysis of the movies began at 2hpi, as this is approximately the amount of time required for the seeded MeWo cells to attach. The analysis revealed that once initiated, the appearance of GFP in the axons was a rapid process, requiring only a few minutes to partially fill axons with GFP for a distance of tens of microns. The fluorescence intensity in the axons was graded, with the signal greatest adjacent to the infecting cells during first hours after infection (bottom of the images), with the intensity dropping as the axons approach the channels connecting the axonal compartment with the cell-bodies compartment (top of images). These observations are consistent with transfer of cytoplasmic components of VZV-infected MeWo cells directly to axons.

### VZV-encoded proteins rapidly appear in axons contacted by VZV-infected non-neuronal cells

**Fig 3:**
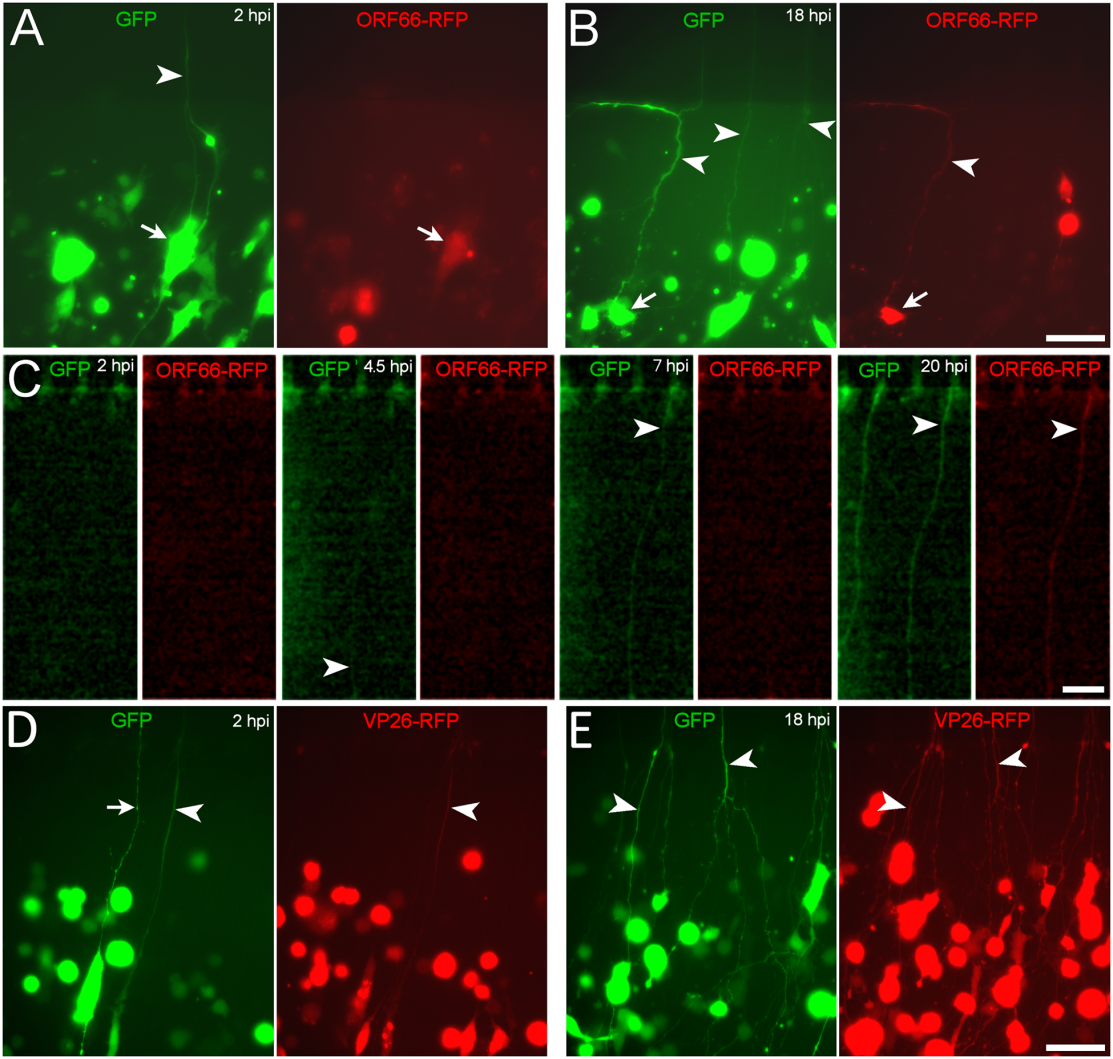
Transfer of both viral and non-viral encoded proteins from alphaherpesvirus-infected MeWo cells to axons. (A–C) MeWo-GFP cells were infected with cell-free VZV66RFP 1 day prior to their plating into the axonal compartment. Pairs of images of microscopic fields of living cultures visualized with GFP filters on the left, and RFP filters on the right. (A) A 2 hpi, a GFP-filled axon (arrowhead) tightly associated with an ORF66-RFP-expressing MeWo cell (arrow) was visualized in the axonal compartment. No RFP fluorescence was observed in axons at 2 hpi. By 18 hpi (B), GFP-filled axons were observed in the axonal compartment (arrowheads), and some were also filled with ORF66-RFP (arrowheads). A MeWo cell in contact with a double-fluorescent axon is indicated by the arrow. (C) Time series of images of a field adjacent to microfluidic channels (top) of another axonal compartment infected with VZV66RFP-infected, MeWo-GFP cells. The first GFP positive axon appeared about 4 hpi (arrowheads). Again, ORF66-RFP appeared in the axons much later (arrowhead). (D&E) HSV-1 infected MeWo cells transfer proteins to axons. HSV-VP26RFP-infected MeWo-GFP cells were plated into axonal compartments as in A & B. At 2hpi some green and red double-fluorescent axons were observed (arrowhead). At 18hpi many GFP and RFP-positive axons (arrowheads) were present in the axonal compartment. Scale bars: 50μm.

We then asked whether VZV-encoded proteins would also appear in axons contacted by VZV-infected non-neuronal cells. MeWo-GFP cells (MeWo cells that constitutively express GFP), were infected with cell-free VZV66RFP and plated in axonal compartments of microfluidic chambers. The infecting MeWo cells thus expressed both ORF66RFP encoded by the virus and GFP encoded by the MeWo cells. GFP was observed in axons by 2 hpi (Fig 3A) but RFP-positive axons were not observed at this time point. However, by 18hpi, GFP/RFP double-fluorescent axons were found in the axonal compartment (Fig 3B). Time-lapse imaging confirmed that both GFP and ORF66RFP appeared in the axons, with the green fluorescence preceding the red fluorescence (Fig 3C). As mentioned above, this filling was infection-dependent, since fluorescent axons never appeared in axonal compartments seeded with uninfected MeWo-GFP cells. This result demonstrates that both non-viral proteins (GFP driven by an independent, non-VZV promoter) as well as VZV-encoded proteins appear in axons after contact with VZV-infected non-neuronal cells.

The closely-related alphaherpesvirus HSV1 also infects axons in vitro (i.e. [19–20] reviewed in [21]). We therefore asked whether plating fluorescent HSV-1-infected non-neuronal cells in axonal compartments would result in the appearance of fluorescence in axons. HSV-VP26RFP-infected GFP-expressing MeWo cells were plated into axonal compartments of microfluidic chambers and monitored for 24 hours. As observed for VZV, GFP+ axons were observed by 2hpi (Fig 3D). However, in contrast to VZV, some of the axons were also RFP fluorescent at this time point. This may be due to the smaller size of VP26mRFP compared to ORF66 (12KDa vs 44KDa for the native proteins, and see section on FRAP analysis below). Notably, 2 hours is much too short a period to allow retrograde axonal transport of HSV-1, infection of the cell bodies and production and diffusion of viral proteins back to the axonal compartment. This result demonstrates that cytoplasmic components of non-neuronal cells infected with another neurotropic alphaherpes virus also appear in co-cultured axons at a time too early to be explained by retrograde infection and synthesis of proteins in the neuronal cell bodies. By 18hpi, many more GFP and RFP-positive axons were present in the axonal compartment (Fig 3E), with the vast majority showing dual florescence. Significantly, even at 18 hpi, no RFP fluorescent neuronal cell bodies were observed in the cell-body compartment of the chambers. Therefore, the source of the RFP observed in the axons could not have been the result of synthesis of VP26 in the neurons and diffusion out to the axons, and the RFP must have been synthesized in the axonal compartment.

**Fig 4:**
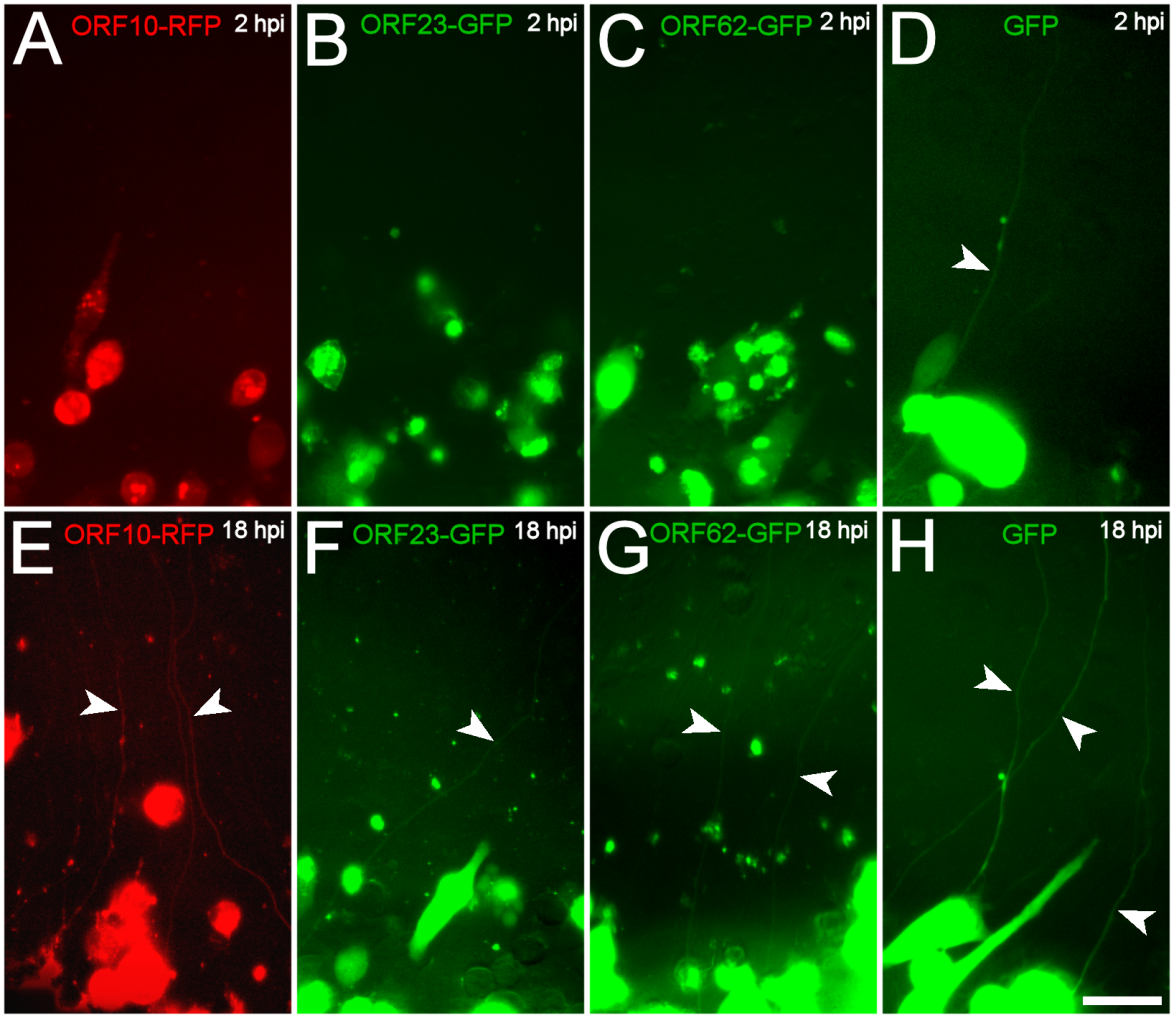
Multiple VZV-encoded proteins are transferred from VZV-infected MeWo cells to axons. MeWo cells infected with VZV-ORF10-RFP (A&E), VZV-ORF23-GFP (B&F), VZV-ORF62-GFP (C&G) and VZV-GFP (D&H) cells with were seeded into the axonal compartments of the microfluidic chambers and images were taken at 2 and 18 hpi. All of the fluorescently-tagged viral proteins filled the axons in the axonal compartments at 18, but not 2 hpi (arrowheads). Scale bar: 50 μm.

In order to determine if other VZV-encoded proteins also appeared in axons after cell-associated infection, we seeded MeWo cells infected with several fluorescent fusion protein-expressing VZV (VZV10RFP, VZV23GFP, VZV62GFP and VZVGFP) into the axonal compartments of microfluidic chambers (Fig 4). ORF10 and ORF62 are tegument proteins, like ORF66, and ORF23 is a capsid protein. At 2hpi axonal filling by fluorescent proteins was only observed for the VZVGFP virus, whose GFP synthesis is driven by an independent SV40 promoter and is not a fusion with a VZV protein. However, at 18 hpi (again, a time point before we have ever observed fluorescent neuronal somata in these experimental conditions), axons in all the chambers contained diffuse fluorescence, demonstrating that ORF10, IE62 and ORF23 proteins like ORF66 appeared in axons after co-culture with VZV-infected non-neuronal cells. It should be noted that axons in chambers containing input cells infected with all three viruses that are fusions to VZV proteins, displayed both diffuse GFP fluorescence and fluorescent puncta at 18hpi. This is because ORF23 is a capsid protein, and both ORF10 and IE62 are components of the inner tegument assumed to be transported along with the capsid (Grigoryan et al, unpublished).

### Appearance of proteins in axons after co-culture with VZV-infected non-neuronal cells is likely due to fusion

**Fig 5:**
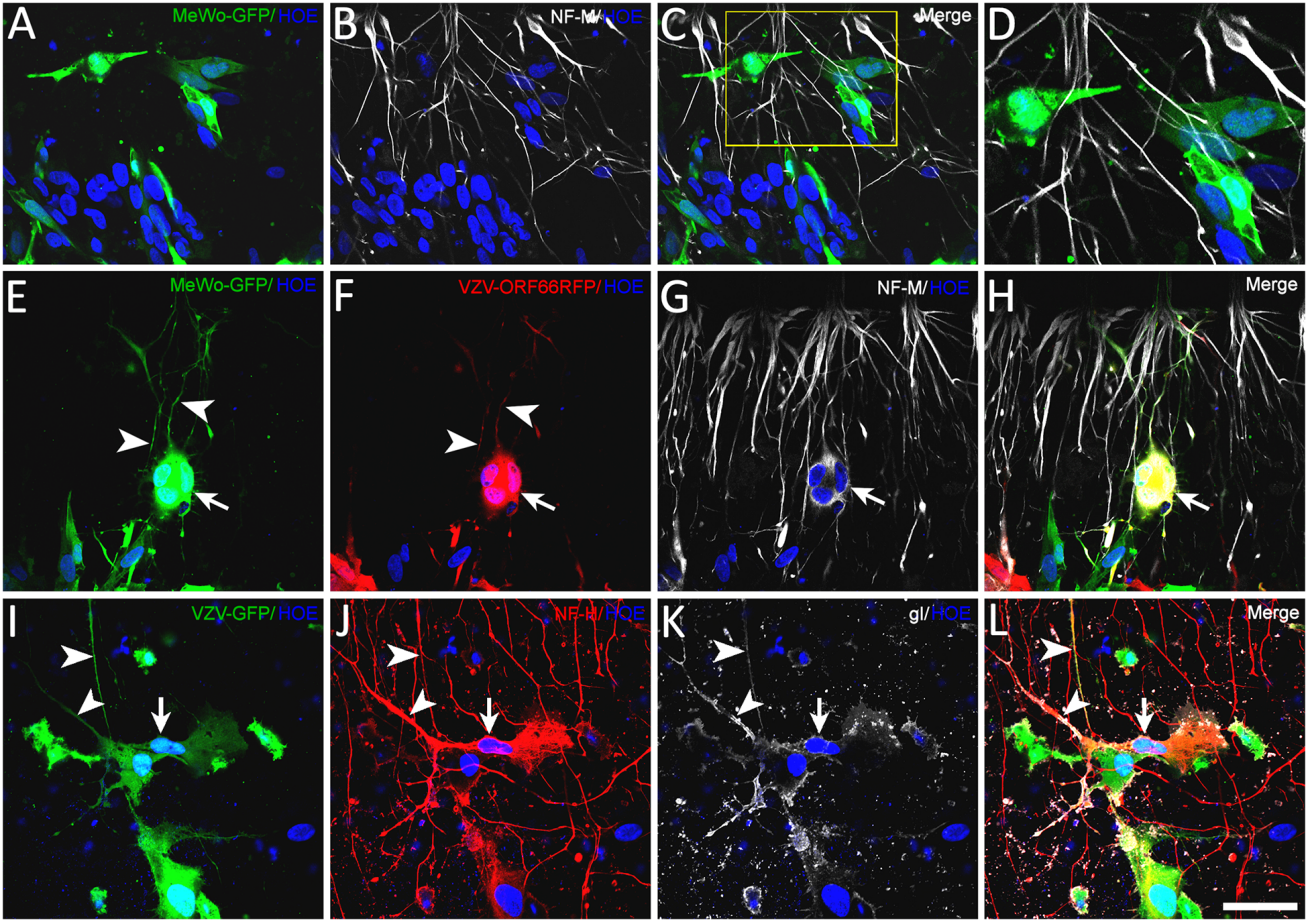
Immunofluorescence evidence of fusion of VZV-infected MeWo cells with axons. MeWo-GFP cells (green) infected with cell-free VZV66RFP (red) virus were plated into axonal compartments of microfluidic chambers or in glass-bottom culture dishes (Fig 1C). Cultures were fixed and immunostained for the medium neurofilament subunit (NF-M, white) 1 to 3 dpi and nuclei stained with Hoechst (blue). (A–D) Control experiments with uninfected MeWo-GFP cells. No GFP was found in the NF-M+ axons. (E–H) Immunostaining of an axonal compartment where MeWo-GFP cells infected with VZV66RFP were plated 3 days prior to fixation. (E&F) Images showing GFP and ORF66RFP fluorescence, respectively. The arrow points to a syncytium of MeWo cells containing both fluorescent proteins. Arrowheads point to axons showing GFP and RFP signal. (G) shows immunocytochemical staining of this field for NF-M, the syncytium observed in E and F to contain both GFP and RFP is NF-M+ as well (white). H shows a merge of all channels, where both white (NF-M) and yellow (co-expression of GFP and RFP) staining are present in the same polykaryon. (I–L) An axonal compartment with VZV-GFP-infected ARPE cells (green) was immunostained at 4 dpi with anti-NF-H 4142 (red) and anti-VZV gI (white) antibodies. I shows GFP fluorescence and J, the immunostaining for NF-M. Many GFP+/NF-M+ axons (arrowheads) were observed. Some infected ARPE cells (arrow) became neurofilament+ after fusion with axons. The fused ARPE cells and axons were also immunopositive for glycoprotein I (K), but not several other cells that were not part of the polykaryon. Scale bar: 50μm.

The simplest explanation for the appearance of fluorescence in axons after co-culture with infected non-neuronal cells is fusion between the axons and the cells. In order to obtain evidence that VZV-infected MeWo cells and axons fuse, we performed a series of immunostainings with an antibody recognizing the neuron-specific neurofilament-M protein (NF-M) in axonal compartments seeded with MeWo-GFP cells previously infected with cell-free VZV66RFP. As controls, we stained parallel cultures containing axons and uninfected MeWo-GFP cells (Fig 5A–5D). Immunostaining revealed that some RFP/GFP-fluorescent axons were contacted syncytia formed by fusion of VZV-infected MeWo cells (Fig 5E–5H). Strikingly, some MeWo syncytia became immunopositive for cytoplasmic NF-M (Fig 5G, arrowed cell). NF-M immunoreactivity was not observed in uninfected MeWo cells or VZV-infected MeWo cells not associated with axons, and therefore was not the consequence of viral up-regulation of the NF-M gene in these cells (not shown). The presence of NF-M protein in syncytia of MeWo cells is consistent with the fusion of axons with the syncytia. Similar results were obtained using VZV-GFP-infected ARPE19 cells. Syncytia of VZV-infected gI immunopositive ARPE19 cells adjacent to axons were NF-M immunopositive at 4 dpi (Fig 5I–5L), suggesting that multiple VZV-infected non-neuronal cells may be able to fuse with axons.

An alternative potential mechanism for the appearance of fluorescent proteins in axons after co-culture with VZV-infected non-neuronal cells is that viral RNA could somehow enter the axons and be translated locally by axonal ribosomes [22]. In order to evaluate this possibility, the VZV-infected MeWo cells were seeded onto axons in compartmented chambers in the presence of cycloheximide to inhibit protein synthesis and the cultures were observed 1,2,3 and 12 hours after seeding (n = 2 independent experiments). Addition of cycloheximide to the culture medium had no effect on the appearance of GFP in the axons (not shown). Three hours and 12 hours after seeding, similar numbers of GFP+ axons were observed in cycloheximide-treated as in untreated cultures. Therefore, local axonal synthesis of GFP is not responsible for the diffuse filling of axons after incubation with VZV-infected non-neuronal cells. The lack of effect on cycloheximide treatment on the filling of axons with proteins from co-cultured cells also demonstrates that viral replication, which is dependent on protein synthesis, is not required for the apparent transfer of fluorescent proteins from infected non-neuronal cells to axons.

### Neurons are incorporated into syncytia and fuse with VZV-infected non-neuronal cells

**Fig 6:**
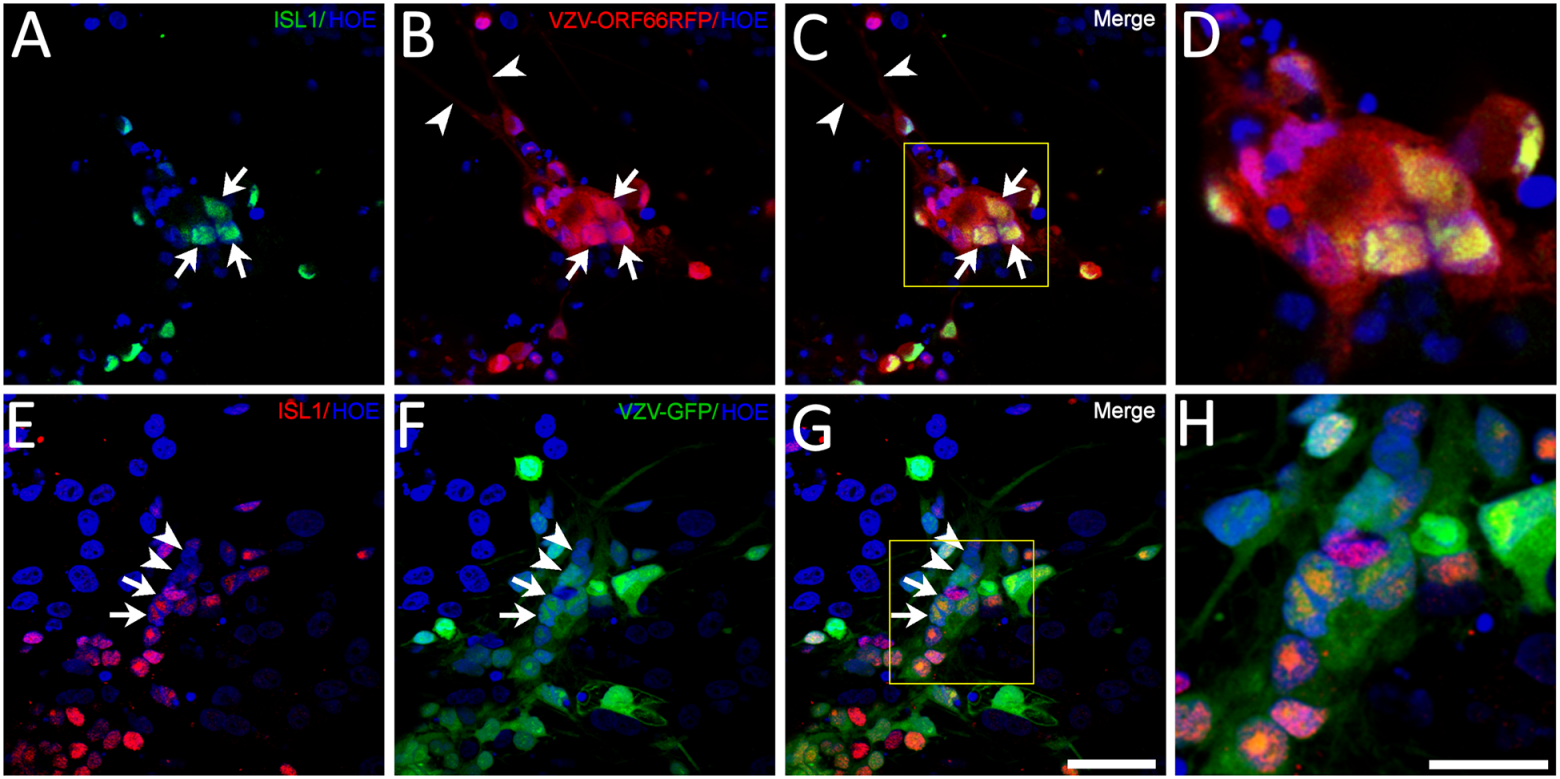
Immunocytochemical evidence for fusion of neurons with VZV-infected non-neuronal cells. (A–D) VZV66RFP-infected MeWo cells plated onto neurons grown in conventional tissue culture immunostained at 3dpi for Islet (Isl1), a neuronal nuclear marker. Isl-1+ green nuclei (arrows) were present inside an RFP-fluorescent polykaryon (B) (arrows). Arrowhead indicates an RFP+ axon. A merged image of RFP and Isl-1 staining is seen in C, and a higher magnification of the portion of the image framed in C is shown in D. In an independent experiment, VZV-GFP-infected MeWo cells were plated onto neurons on coverslips, and were immunostained for Isl-1 (E–H). Again, many Isl-1+ (red, arrows) nuclei were observed as part of GFP+ (green) polykaryon. A few adjacent Isl-1- nuclei that are part of the polykaryon are indicated by the arrowheads. Axonal GFP expression was relatively weak due to the images being thin optical sections with the confocal microscope. Nuclei stained blue with Hoechst 33258 dye. Scale bars: A-C, E-G -50μm; D and H—25μm.

Studies of fixed fetal dorsal root ganglia transplanted to SCID mice suggested that neurons and satellite cells infected with VZV fuse to form syncytia [1]. We therefore asked whether VZV-infected non-neuronal cells can fuse with neuronal cell-bodies in our in-vitro system in addition to fusing with axons. Neuronal cultures were fixed 3d after addition of VZV66RFP-infected MeWo cells and stained for the for the nuclear transcription factor Isl-1 (Fig 6). IsI-1 is normally expressed by sensory and sympathetic neurons [23–24] present in our cultures, as well as spinal motoneurons and pancreatic endocrine cells that are not found in our cultures. Isl-1+ nuclei were observed within syncytia that formed from VZV66RFP-infected cells (Fig 6A–6D). Similar results were obtained using MeWo cells infected with VZV-GFP, syncytia including Isl-1+ nuclei were present in the cultures (Fig 6E–6H). VZV-infected MeWo cells that were not part of syncytia never contained Isl-1+ nuclei.

In order to confirm that non-neuronal cells can undergo fusion with human neurons, we mixed uninfected MeWoGFP cells with VZV66RFP-infected ARPE cells and seeded them into neuronal cultures (a diagrammatic representation of the experiment is shown in Fig 1C) and followed events in the cultures using live imaging. Syncytia initially formed between VZV-infected ARPE cells, and MeWo cells subsequently fused into the growing polykarya (S2 Movie). The mixing of the cytoplasmic contents of the ARPE and MeWo cells was easily detected in the movies by the change in color of the fluorescence in the syncytia from red to yellow. Cells with neuronal morphology (phase bright cell body and fine neurites) appeared to join the yellow-fluorescent polykarya. These syncytia eventually included most of the cells in the dish, with all the cells dying by 3–4 dpi. These results from the immunostaining and the live imaging experiments strongly suggest that neurons fuse with non-neuronal cells in co-cultures.

### FRAP studies of fluorescent protein diffusion in and transfer to axons

**Fig 7:**
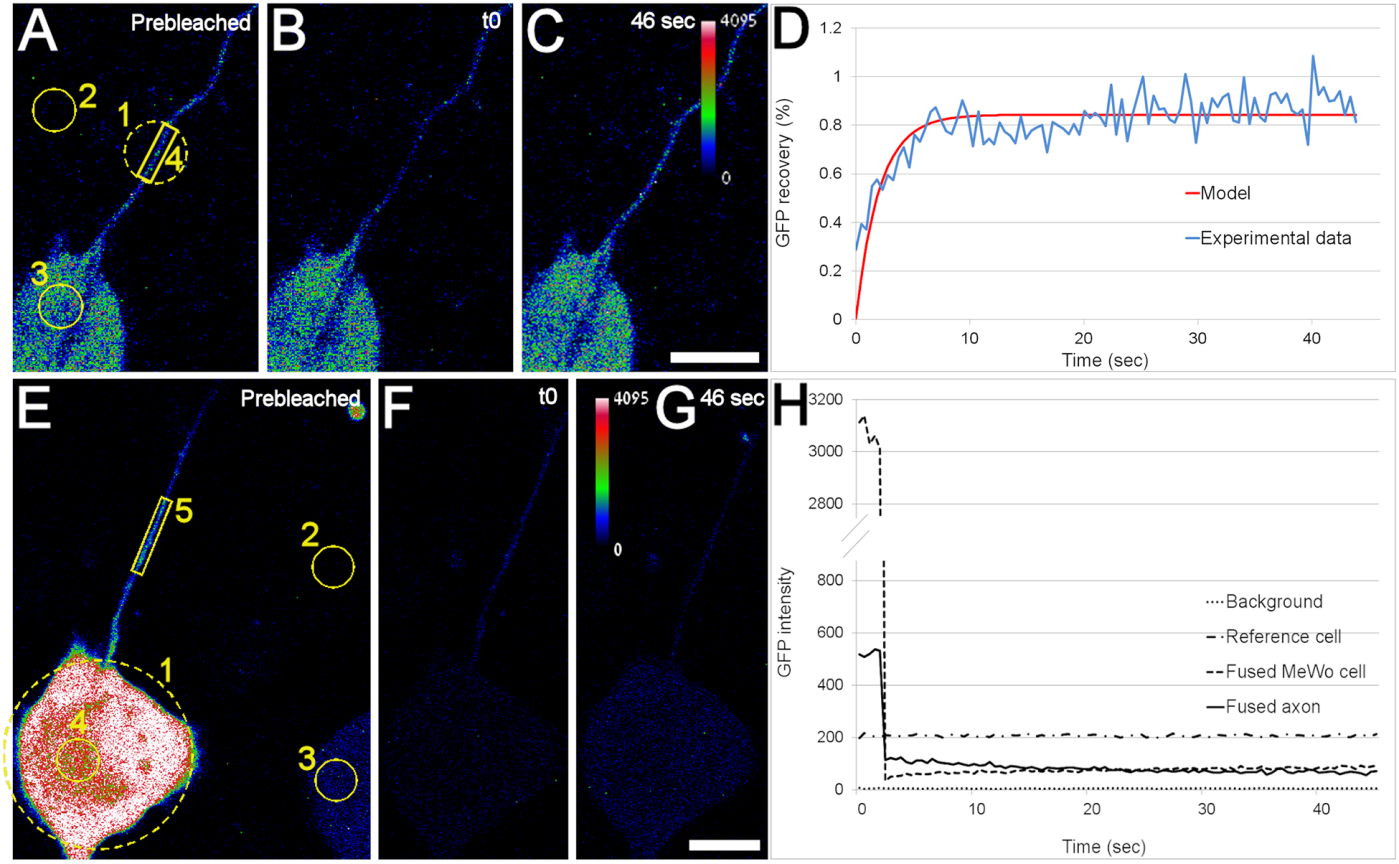
Transfer of protein from VZV-infected cells to axons studied using fluorescence recovery after photobleaching (FRAP). A–D Measurement of viral and non-viral protein diffusion coefficients in axons using FRAP 1–2hpi. VZV-GFP or VZV66RFP-infected MeWo cells were plated into axonal compartments of microfluidic chambers and the first fluorescent axons were imaged using a confocal microscope. A–D depict a FRAP analysis of a GFP-positive axon. (A) A MeWo cell and an axon before bleaching shown using an RGB-intensity look up table (LUT). Circles and polygons showing the areas that were bleached and measured: 1—bleached area, 2—area measured for determining background level, 3—area used as reference to the level of fluorescence in the input MeWo cell, 4—area measured to determine axonal fluorescence recovery. (B) The axon immediately after photobleaching (t0). (C) The same axon at the end of the recovery period. The fluorescence level recovered to a level very similar to that before bleaching. (D) The observed fluorescence recovery curve (blue) and the fitted model curve (red) of the axon shown in (A–C). The close fit of the model yields an estimate of the diffusion coefficient of the fluorescent molecule. Similar results were obtained from 10 axons where the RFP or GFP was bleached and measured. (E–H) Further evidence of diffusional transfer of fluorescent protein from non-neuronal cells to axons using FRAP at 1 to 2 hpi. An entire VZV-infected MeWo-GFP cell was photobleached (circle 1) and the GFP intensities were measured in the cell (circle 4) and the neighboring GFP-filled axon (polygon 5) for about 45s. Fluorescence intensity of the background (circle 2) and that of another MeWo cell (circle 3) were also measured. (F) The MeWo cell and the axon immediately after bleaching for 1sec (t0). (G) The same area after 46s of recovery. In contrast to the result shown in (C) above, the GFP fluorescence in the axon did not recover. (H) Graphical representation of fluorescence intensities in the MeWo cell (dashed curve) and the adjacent axon (solid line). The lack of fluorescence in the axon and the lack of recovery were apparently due to both the diffusion of GFP in the axon away from the visualized portion, and the lack of replenishment of GFP due to the bleaching of the GFP in the adjacent MeWo cell. Scale Bars: 10μm.

As mentioned above, when VZV-66RFP-infected MeWoGFP cells were used to infect axons, GFP fluorescence was observed in axons earlier than ORF66RFP. ORF66RFP has a molecular mass of approximately 75KDa, compared the 25KDa mass of GFP. Therefore, we suspected that the delay in the appearance of red fluorescence in the axons was due to slower diffusion. In order to test this hypothesis, we utilized fluorescence recovery after photobleaching (FRAP) to estimate the diffusion coefficients of GFP and ORF66-RFP in axons (see Materials and Methods, Fig 7A–7D). We estimated the diffusion coefficient of GFP in MeWo cells to be 27 μ^2^/sec, consistent with that reported by others [25] (Table 1). We calculated the diffusion coefficient of GFP in axons to be 14 μ^2^/sec, much lower than that in MeWo cells. This might be due to the denser cytoskeleton in axons that is required for fast retro- and anterograde axonal transport or the narrow dimensions of axons. The diffusion coefficient of ORF66RFP in axons was calculated to be 6.6 μ^2^/sec, less than half of that of GFP. These calculations support the hypothesis that the more rapid filling of axons with GFP than ORF66RFP was due at least in part to size, since the larger viral fusion proteins showed slower diffusion than GFP.

**Table 1:** Diffusion coefficients of GFP and ORF66-RFP in axons and MeWo cell cytoplasm measured by FRAP.

	GFP in axons	GFP in MeWo cell cytoplasm	ORF66-RFP in axons	ORF66-RFP in MeWo cell cytoplasm
Diffusion coefficient (μm2/s)	14 (n = 10)	27 (n = 13)	6.6 (n = 10)	5.6 (n = 13)

Diffusion coefficients of GFP and ORF66GFP in axons and MeWo cells measured using FRAP as described in the methods and in the legend to Fig 7.

Our hypothesis of direct transfer of proteins from non-neuronal cells to axons was also supported by FRAP experiments in which whole VZV-infected MeWo cells found in physical interaction with axons were photobleached. A significant decrease in GFP intensity in axons was observed after photobleaching of adjacent cells due to the diffusion of GFP in the axon and lack of a de novo source of GFP (Fig 7E–7H). Axons adjacent to VZV66RFP infected MeWo cells that were photobleached also showed weaker fluorescence (not shown), confirming that not only free non-viral proteins (GFP), but also diffuse viral proteins (ORF66-RFP) are likely directly transferred from input cells to axons. Since VZV is a fusogenic virus, and neurons and non-neuronal cells are incorporated into common syncytia, it is likely that the transfer of protein from non-neuronal cells to axons is also due to fusion of axons with the infected cells.

## Discussion

Cell-to-cell fusion induced by VZV infection has long been known to occur among fibroblasts and keratinocytes during the formation of vesicles in the skin, during both varicella and zoster. It is not known, however, whether neurons also fuse with other cells in VZV disease. Examination of VZV-infected human DRG in the SCID-hu model of VZV neuropathogenesis suggested that neurons can undergo fusion with surrounding satellite cells [26]. We describe here the VZV-infection-dependent passage of cytoplasmic proteins, both of viral and non-viral origin, from VZV-infected cells to human axons during cell-associated infection. Diffuse GFP was observed in axons in axonal compartments of compartmentalized microfluidic chambers by 2 hours after plating input cells expressing GFP driven by a non-VZV promoter. Four different fluorescent fusions of VZV-encoded proteins were observed in axons by 18 hpi. The fluorescent VZV-encoded observed proteins could not have been synthesized by the neuronal cell bodies and transported to the axons, since fluorescent neurons were never observed in the cell body compartments of the chambers in these (and previous [9],[12]) experiments at this time point.

Another mechanism that could possibly explain the bulk transfer of proteins from non-neuronal cells to axons would be via exosomes, which are induced upon viral infection of cells (reviewed in [27]). However, our observation that neuronal cell bodies apparently join syncytia of VZV-infected non-neuronal cells is most simply explained by VZV being fusogenic for neurons and their processes. This hypothesis is consistent with observations from embryonic DRG transplanted to SCID mice showing lack of membranes separating between neurons and satellite cells in VZV-infected ganglia [1]) indicating fusion with adjacent non-neuronal cells. Generation of “fusion pores” (or possibly syncytia) between neurons infected with the related alphaherpesvirus PRV in culture has also been demonstrated by flow of fluorescently-labeled dextran between neurons [28]. Future experiments using VZV-mutants for glycoproteins involved in syncytia formation may reveal which of these is responsible for fusion of non-neuronal cells with axons.

There have been several studies of retrograde infection of axons by other fluorescent alphaherpesviruses using experimental apparati that separate axons from their somata (i.e. [19], [29]. While diffuse filling of axons with fluorescent proteins was not reported in these studies, it should be noted that most studies of PrV and HSV use cell-free viral preparations, where there are no input cells to fuse with the axons. When we infected axons with HSV1 in a cell-associated manner using compartmentalized microfluidic chambers in the present study, we indeed observed the rapid transfer of fluorescent protein to the axons, raising the possibility that this occurs in HSV infections in-vivo as well. This raises the intriguing question as to why such fusion events do not apparently occur among cells in fetal human DRG transplanted to SCID mice when infected by HSV infection [30]. It is possible that HSV capsids are primarily exported into axons and the most final assembly of infectious virions is at the distal end of axons rather than in the ganglion, which would prevent much fusion from taking place in the ganglia.

Upon axonal infection in compartmentalized chambers, VZV nucleocapsids are rapidly transported to neuronal cell bodies at a rate similar to that reported for HSV and PrV [12]. If VZV-infection of cutaneous axons involves fusion, it is possible that VZV nucleocapsids in the input cell cytoplasm may enter axons directly, in addition to or instead of fusing with the plasma membrane and being stripped of the viral envelope and outer tegument proteins as occurs with HSV and PRV [31]. Preliminary experiments using VZV doubly-labeled with fluorescently outer tegument and capsid proteins show that most capsids do not co-transport with outer tegument proteins (Grigoyan et al, in preparation). These observations are consistent with nucleocapsids and not intact virions being transported to the somata using fast axonal transport as has been shown for other herpesviruses in-vitro (reviewed in [32–33]).

The diffusion of VZV proteins from input cells to axons in cell-associated infection in vitro may explain a discrepancy between our results using VZV and observations of HSV1 infection. It was recently reported that when HSV1 infects axons in vitro, it predominantly gives rise to a silent infection [19]. The authors in that study suggested that the reason for the lack of a productive HSV1 infection in their experimental system may be due to insufficient levels of VP16 protein as originally proposed by Roizman and Sears ([34] cited in [19]). By contrast, in our experiments using VZV, we almost always obtain a productive infection following axonal infection that subsequently spreads among the neuronal somata ([9], [12]). This may be the consequence of fusion mediated entry of ORF10, the VZV homolog of the HSV VP16 IE transcriptional transactivator, to axons infected in a cell fusion-associated manner. In order to estimate the time for diffusion of ORF66RFP into neuronal somata, we calculated the typical first passage time for a diffusing molecule in a one dimensional tube. We assumed a source at the axonal contact with the infecting cell to be an infinite absorption boundary. Solving this one-dimensional diffusion problem, the typical time for arrival of a molecule at the neuron is given by: τ = Δx^2^/6D. In our experimental setup with approximately 1 mm long axons and a diffusion coefficient of about 6 μm^2^/s, we estimated that proteins the size of ORF66RFP should reach the neuron by diffusion after about 8 hours. This means with cell-associated infection that we use, high levels of similarly sized ORF10 probably arrive in a timely manner to the neuronal somata that leads to a productive infection, rather than entry into a silent (latent-like) infection. Indeed, recent experiments in our laboratory infecting axons with cell-free VZV result in a silent infection with long-term retention of VZV genomes and no productive infection (Markus et al, submitted). It remains possible, however, that some tegument proteins reach the cell body more rapidly by either being taken up into vesicles or binding cytosolic proteins that associate with the rapid axonal transport machinery of the neuron. This would allow these proteins to arrive in the soma more rapidly and therefore play a role in the decision between latent and productive infection.

Our in-vitro results suggest that in addition to neural fusion events in the ganglion [1], axons of sensory neurons innervating the skin may fuse with syncytia of fibroblasts and/or keratinocytes that surround varicella and zoster vesicles in during disease. These fusion events have potential clinical implications in VZV disease. In VZV (and HSV) skin lesions during primary disease, there are large quantities of free virus present in the vesicular liquid [8]. Thus, we speculate that there may be multiple modes of retrograde neuronal infection via peripheral axons. The “classical” model is where virus enters peripheral axons by endocytosis or fusion, has its membrane and outer tegument layers stripped off, to expose the inner tegument proteins that function to allow transport of the nucleocapsid within the axons to the nucleus of the neuron. An alternative model is that axons could fuse with virus-infected skin cells, and in addition to nucleocapsids, tegmental proteins being synthesized by the fusing cell would be transferred en masse into the axons. This would presumably transfer much higher levels of protein than associate with the tegument of individual infecting virions. Unassisted diffusion of proteins from the skin to the trigeminal or dorsal root ganglion would take many weeks but such proteins might be transported to the soma by rapid transport systems in parallel to the nucleocapsids, where they could participate in establishing the initiation of a productive infection of the neuron. In varicella, most retrograde infection of ganglionic neurons from the skin results in a latent infection. This axonal infection is therefore likely by cell-free virus infection of the classical type described above where tegmental proteins critical for viral replication are presumably not transported to the soma rapidly.

After reactivation of latent VZV in zoster, infectious virus is made in neuronal somata (and possibly spread by fusion with wrapping glial cells [1]) and transported along axons to the periphery. Fusion between axons and skin cells from this anterograde infection would change the membrane characteristics of axons and could result in increased firing in nociceptors. Abnormal electrophysiological properties are indeed observed when PRV-infected neurons fuse with one another [28]. Such abnormal electrophysiological activity could play a role in the generation of neuropathic pain that often accompanies herpes zoster. The hESC-derived neuron model with fluorescent VZV used here could be used to examine the electrophysiological consequences of axonal or somatic fusion using voltage sensitive dyes, or conventional electrophysiological recordings.

## Supporting Information

S1 Movie Rapid transfer of protein from VZV-infected MeWo cells to axons. Fig 2 was made from individual frames of this time-lapse movie. Four axons are observed to fill with GFP in this movie. The acquisition of the movie was initiated 2h after seending the MeWo cells. The rate of image capture was one frame/5 min. Scale bar: 50μm.
(AVI)

S2 Movie Live imaging of VZV-infection dependent syncytium formation of neurons with two types of non-neuronal cells. VZV-66RFP infected ARPE cells and uninfected MeWo-GFP were mixed and plated on differentiated neurons in glass-bottom dishes (Fig 1C) and monitored starting 5 hours after plating for 72 hours. VZV-ORF66-RFP infected ARPE cells (red), MeWo-GFP cells (green) and neurons (phase bright small cells with long processes at arrows) fuse into at least two syncytia that continue to grow and spread out with time. After three days of observation, the syncytia have disintegrated as the cells die. The rate of image capture was one frame/30min Scale bar: 25μm.
(AVI)
